# A High‐Entropy Strategy for Chemoresistive Ethanol Sensors With Remarkably Rapid and Selective Response

**DOI:** 10.1002/advs.75724

**Published:** 2026-05-20

**Authors:** Gi Baek Nam, Jin Ho Seo, Youngmin Kim, Hyuk Jin Kim, Yeong Jae Kim, Seon Ju Park, YunKyung Kim, Hyeon‐Min Yu, Jiwoo Lee, Ji Hyeon Lim, Jiheon Lim, Jong Hun Kang, WooChul Jung, Jong Jin Jung, Jeong Woo Han, Seung Eon Moon, Seong Ju Hwang, Jong‐Hoon Kang, Zhigang Zhu, Ho Won Jang

**Affiliations:** ^1^ Department of Materials Science and Engineering Research Institute of Advanced Materials Seoul National University Seoul Republic of Korea; ^2^ School of Chemical and Biological Engineering and the Institute of Chemical Process Seoul National University Seoul Republic of Korea; ^3^ Sentech Korea Corp. Gyeonggi Republic of Korea; ^4^ Smart Materials Research Section Electronics and Telecommunications Research Institute Daejeon Republic of Korea; ^5^ Department of Materials Science and Engineering Pohang University of Science and Technology (POSTECH) Pohang Republic of Korea; ^6^ Center for Van der Waals Quantum Solids Institute For Basic Science (IBS) Pohang Republic of Korea; ^7^ School of Health Science and Engineering University of Shanghai for Science and Technology Shanghai China; ^8^ Advanced Institute of Convergence Technology Seoul National University Suwon Republic of Korea

**Keywords:** ethanol, gas sensors, high‐entropy oxide, nanostructure, solvothermal synthesis

## Abstract

Chemoresistive gas sensors based on metal oxide semiconductors provide a stable and low‐cost platform for gas monitoring. However, single‐metal oxides exhibit limited sensitivity and selectivity due to insufficient active sites and weak catalytic activity. Although metal doping and noble‐metal decoration provide partial improvements, these strategies remain constrained by dopant instability, restricted compositional diversity, and high material costs of noble metals. High‐entropy materials offer an attractive platform for tuning structure and reactivity through large configurational entropy, which induces lattice distortion, diverse electronic coordination, and defect‐rich environments. Here, we present an ethanol (C_2_H_5_OH) gas sensor based on high‐entropy oxide (HEO) nanostructures composed of In, Sn, Fe, Zn, and W. The HEO‐based sensor exhibits a higher response to ethanol than low‐ and medium‐entropy oxides and maintains fast kinetics, stable cycling, and reliable operation under humid conditions. Mechanistic analysis reveals that entropy‐driven shifts in the *d*‐band structure, the enrichment of oxygen vacancies, and increased chemisorbed oxygen strengthen the surface reaction pathway. These findings establish configurational entropy as an effective strategy for chemoresistive gas sensors with high reactivity and robust long‐term performance.

## Graphical abstract

The configurational‐entropy strategy enables rapid and sensitive ethanol detection using a multicomponent oxide. The high‐entropy (InSnFeZnW)O_x_ provides enriched oxygen vacancies and diverse adsorption sites that strengthen surface reactivity. The sensor achieves a 5135% ethanol response with fast kinetics and stable long‐term operation.

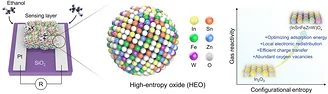

## 1 Introduction

The rapid development of artificial intelligence (AI) and Internet of Things (IoT) technologies has accelerated the demand for high‐performance chemical sensors compatible with digital systems [1, 2]. Among various sensing platforms, chemoresistive gas sensors have emerged as one of the most promising candidates because of the facile electrical readout, simple architecture, scalability into sensor arrays, and low fabrication cost [3]. In particular, metal oxide semiconductor (MOS)‐based sensors such as WO_3_ [4], In_2_O_3_ [5], and SnO_2_ [6] have been investigated owing to their chemical stability, reliability, and fast response and recovery. However, single‐metal oxides suffer from limited sensitivity and poor selectivity due to the restricted number of active sites and lack of catalytic effect. To overcome the intrinsic limitations of single‐metal oxide, strategies such as cation doping (e.g., Bi‐In_2_O_3_ [7, 8], Fe‐doped SnO_2_ [9]) and noble‐metal decoration (e.g., Pd‐WO_3_ [10, 11], Au‐SnO_2_ [12, 13]) have been developed to promote catalytic activity and surface reactivity. Although these approaches enhance response magnitude, they still face challenges of structural instability, limited compositional tunability, and high cost associated with noble metals [14].

High‐entropy materials (HEMs), comprising five or more principal elements incorporated into a single solid phase, have recently emerged as a promising class of functional materials for the aerospace industry [15, 16], biomedical engineering [17, 18], and catalysis [19, 20]. The incorporation of multiple principal elements produces diverse atomic environments and variable charge states, leading to structural disorder, a wide range of bonding configurations, and dynamic electron‐exchange pathways [21]. The entropy‐driven characteristics enrich the accessible adsorption sites, facilitate defect formation and migration, and broaden the electronic states that contribute to gas adsorption and surface redox reactions [22]. Recent studies have explored high‐entropy transition‐metal oxides and alloys as chemoresistive sensing materials. For instance, Lee et al. and Wang et al. reported high‐entropy alloy–decorated MOS sensors to replace noble‐metal catalysts, while Rui et al. fabricated a SnZnNiCoCu high‐entropy oxide for acetone detection [23, 24, 25]. Despite these advances, the role of configurational entropy in gas adsorption, defect‐mediated charge transport, and sensing response remains unclear.

**FIGURE 1:**
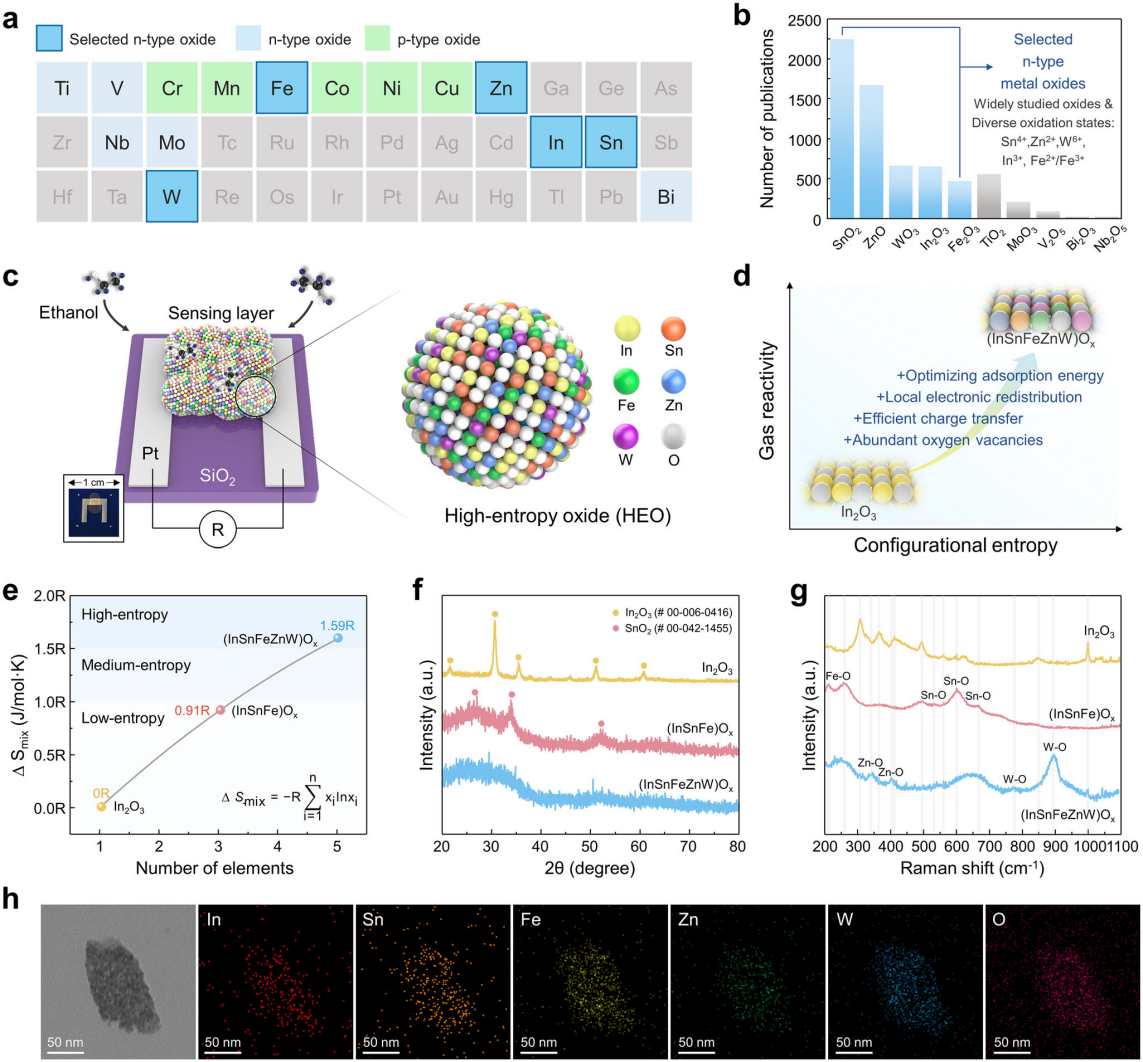
Conceptualization and characterization of high‐entropy oxide (HEO) gas sensors. (a) Periodic table of cation candidates for chemoresistive metal‐oxide gas sensors. (b) Number of publications of n‐type metal oxide gas sensors based on hydrothermal and solvothermal synthesis, with data obtained from the Web of Science Core Collection Search: “TS = (gas sensor*) or (gas detection*) including (MO) using the keywords. (c) Schematic illustration of the HEO‐based ethanol sensor. The inset is the optical image of the sensor. (d) Conceptual diagram showing the enhanced gas reactivity of (InSnFeZnW)O_x_ compared with In_2_O_3_. (e) Configurational entropy versus the number of constituent elements, highlighting transition from low‐ to high‐entropy oxide. (f) XRD patterns and (g) Raman spectra of In_2_O_3_, (InSnFe)O_x_, and (InSnFeZnW)O_x_. (h) High‐resolution TEM and STEM‐EDS elemental mappings of (InSnFeZnW)O_x_ HEO.

Here, we report an ethanol (C_2_H_5_OH) sensor based on a high‐entropy oxide (HEO) composed of In, Sn, Fe, Zn, and W cations. Ethanol detection is of great significance in a wide range of applications, including indoor air‐quality management, breath diagnostics [26, 27], food monitoring [28], and industrial process management [29]. To isolate the effects of configurational entropy and avoid additional variables arising from mixed carrier types, the HEO was designed using only n‐type metal oxide cations, excluding p‐type constituents (Figure 1a). Among widely studied n‐type oxides, In, Sn, Fe, Zn, and W were selected as representative cations because of a broad range of oxidation states, thereby enhancing structural and chemical disorder within the amorphous HEO network (Figure 1b) [30]. To elucidate the role of configurational entropy, we synthesized oxide compositions within low, medium, and high‐entropy regimes under identical conditions, enabling a direct comparison of their structural and sensing characteristics. The ethanol sensing performance improved as configurational entropy increased, and the high‐entropy (InSnFeZnW)O_x_ exhibited the highest response and the fastest response kinetics. This behavior aligns with the stronger structural disorder, diverse surface adsorption sites, and higher density of oxygen‐related defects. Along with the enhanced response, the HEO sensor demonstrated excellent repeatability, long‐term stability, and humidity tolerance.

To clarify the origin of the enhanced sensing performance, valence band X‐ray photoelectron spectroscopy (XPS), O_2_ temperature‐programmed desorption (O_2_‐TPD), O 1*s* XPS, and electron spin resonance (ESR) analyses were conducted. The high‐entropy oxide exhibited a stronger electron‐donor character, higher oxygen adsorption capability, and a greater concentration of oxygen vacancies than the lower‐entropy oxides. Such entropy‐driven structural and electronic modifications facilitate more efficient charge transfer processes, accelerate surface reaction kinetics, and ultimately strengthen the chemoresistive response. These findings highlight entropy engineering as one of several promising strategies for advancing chemoresistive gas sensors with high sensitivity, reliable selectivity, and stable operation across diverse environments.

## 2 Results and Discussion

### 2.1 Preparation and Characterization of (InSnFeZnW)O_x_

To investigate the role of configurational entropy in gas sensing, we designed a high‐entropy oxide as the active sensing layer (Figure 1c). Integrating five different metals (In, Sn, Fe, Zn, and W) into a single oxide lattice increases the configurational entropy and stabilizes a compositionally complex structure. As depicted in Figure 1d, these entropy‐driven modifications are expected to optimize adsorption energies, promote charge transfer, and enrich oxygen‐related defect sites, thereby enabling enhanced gas reactivity toward ethanol molecules compared with lower‐entropy oxides [31].

Building upon these concepts, (InSnFeZnW)O_x_ HEO was synthesized via a solvothermal method using equiatomic proportions of the five metal precursors (see Experimental Section for details). The formation of the HEO proceeds through an autocatalytic growth mechanism, wherein gradual thermal decomposition and reduction of metal precursors generate initial nucleation (Figure S1) [32, 33]. These nuclei serve as catalytic centers that accelerate subsequent metal ion reduction and sustain particle growth through a self‐propagating process. This kinetically driven route enables multiple elements to be incorporated into a single‐phase structure without the need for strong reducing agents or high temperatures. Elemental analysis confirms that the HEO exhibits a uniform compositional distribution of all constituent elements throughout the material (Figure S2 and Table S1).

The thermodynamic stabilization of the multicomponent oxide arises from the high configurational entropy, which lowers the Gibbs free energy and suppresses phase segregation [34]. As illustrated in Figure 1e, the configurational entropy of (InSnFeZnW)O_x_ reaches 1.59R, exceeding that of In_2_O_3_ and (InSnFe)O_x_. This higher configurational entropy promotes random cation distribution and drives the formation of a homogeneous single‐phase structure. When combined with the autocatalytic growth process, the entropy‐driven stabilization distinguishes the high‐entropy oxide from medium‐ or low‐entropy oxides that are prone to compositional heterogeneity.

Structural evolution with increasing configurational entropy is observed in the X‐ray diffraction (XRD) analysis (Figure 1f). The low‐entropy In_2_O_3_ exhibits sharp and intense diffraction peaks characteristic of high crystallinity, whereas the medium‐entropy (InSnFe)O_x_ displays broader peaks with lower intensity, reflecting a lower degree of crystallinity and increased lattice distortion. The peak broadening becomes more pronounced in the high‐entropy (InSnFeZnW)O_x_, indicating suppressed long‐range ordering and structural disorder. The loss of crystallinity arises from random incorporation of elements with different ionic radii and oxidation states, which disrupt periodic lattice symmetry and produce a compositionally disordered oxide lattice [35].

Raman spectroscopy provides further insight into the local bonding environments (Figure 1g). The In_2_O_3_ exhibits characteristic vibrational modes associated with In─O coordination. With the incorporation of additional elements, the (InSnFe)O_x_ shows new vibrational features corresponding to Sn─O and Fe─O bonding, verifying the coexistence of multiple metal‐oxygen linkages. In the (InSnFeZnW)O_x_, additional bands associated with Zn─O and W─O coordination emerge, confirming the successful incorporation of Zn and W into the oxide framework. Compared with the In_2_O_3_ and the (InSnFe)O_x_, the (InSnFeZnW)O_x_ exhibits band broadening and shifts arising from local lattice strain and symmetry disruption induced by the random substitution of chemically diverse atoms [35]. These observations confirm the formation of structural disorder and heterogeneous bonding characteristics of a high‐entropy system.

Transmission electron microscopy (TEM) was carried out to investigate the morphology and elemental distribution of the oxides. The (InSnFeZnW)O_x_ presents an irregular porous morphology composed of interconnected aggregates (Figure S3). High‐resolution TEM and elemental mapping in Figure 1h further confirm that In, Sn, Fe, Zn, W, and O are uniformly distributed throughout the oxide, demonstrating homogeneous incorporation of all elements into a single‐phase structure. Additional TEM and elemental analysis of the reference oxide also show uniform elemental dispersion of their respective constituent elements (Figure S4). Importantly, the high‐entropy system maintains such compositional uniformity despite its significantly greater chemical complexity, underscoring the role of configurational entropy in promoting random cation mixing and preventing phase segregation, thereby preserving both structural integrity and compositional homogeneity.

To further examine the physical characteristics of the HEO, nitrogen adsorption‐desorption measurements were performed. The Brunauer‐Emmett‐Teller (BET) surface areas of In_2_O_3_, (InSnFe)O_x_, and (InSnFeZnW)O_x_ were determined to be 62.54, 23.99, and 94.42 m^2^ g^−1^, respectively (Figure S5a–c). The (InSnFeZnW)O_x_ exhibits a relatively high surface area compared to previously reported high‐entropy oxides (Figure S5d). This large surface area reflects a highly accessible porous structure that provides abundant surface sites for gas interaction [36]. Although configurational entropy is not intrinsically correlated with specific surface area, an increase in surface area expands the interfacial contact region between the oxide surface and gas molecules, thereby increasing the availability of adsorption sites.

### 2.2 Entropy‐Driven Local Structural and Electronic Redistributions

**FIGURE 2:**
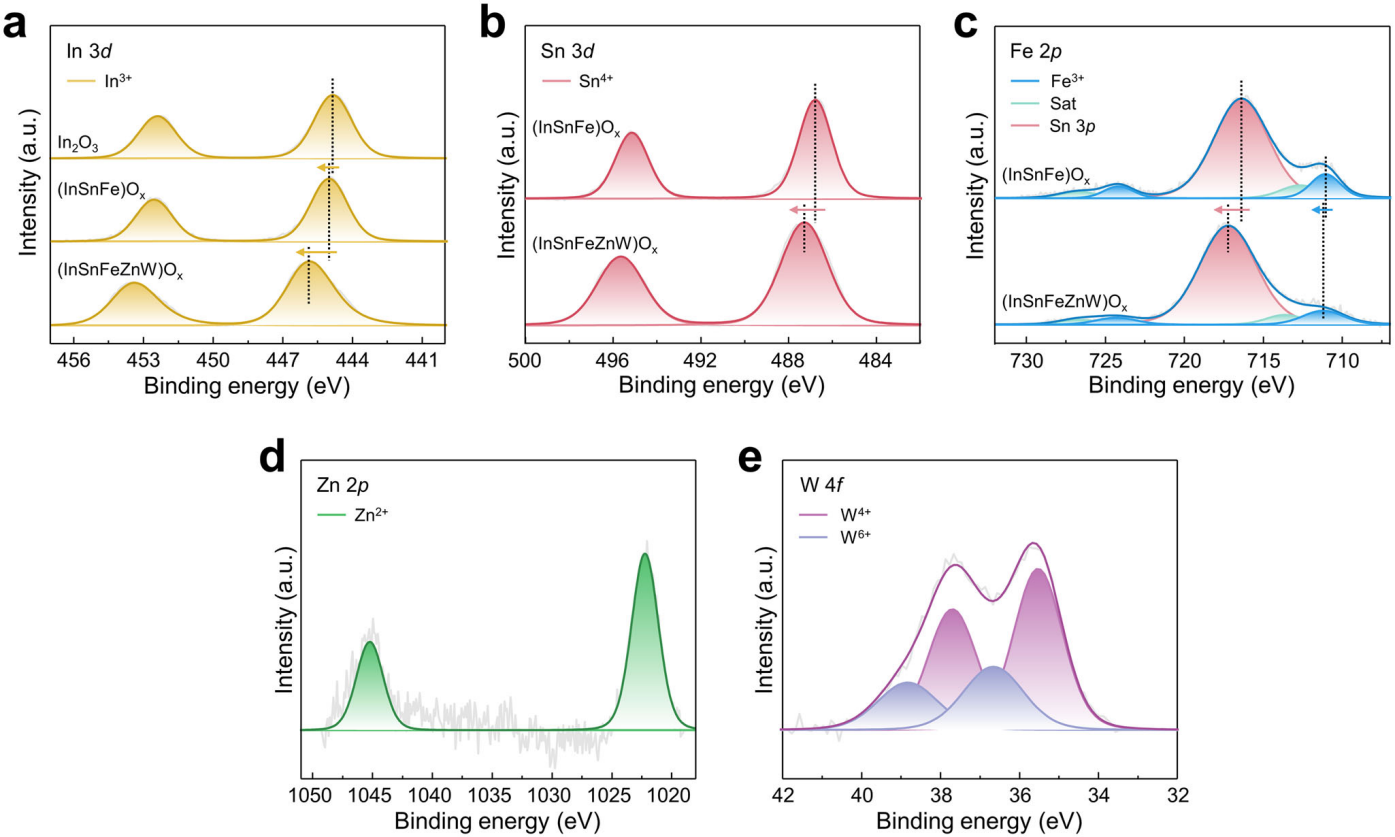
Surface chemical analysis from low‐entropy to high‐entropy oxides. (a) In 3*d*, (b) Sn 3*d*, (c) Fe 2*p*, (d) Zn 2*p*, and (e) W 4*f* XPS spectra of In_2_O_3_, (InSnFe)O_x_, and (InSnFeZnW)O_x_.

X‐ray photoelectron spectroscopy (XPS) was examined to probe the oxidation states and local electronic environments of the constituent metals (Figure S6). In the In 3*d*, Sn 3*d*, and Fe 2*p* regions (Figure 2a–c), the binding energies shift toward higher values as the compositional complexity increases from In_2_O_3_ to (InSnFeZnW)O_x_. This trend indicates electronic redistribution, particularly around In and Sn sites, leading to a more electron‐deficient state of In and Sn in the high‐entropy oxide (Table S2). Consistent with the increased structural disorder associated with higher configurational entropy, these variations reflect modifications in the local bonding environment. The coexistence of elements and bonding preferences perturbs the local charge distribution, and the accompanying lattice distortion amplifies the perturbations [37, 38].

The Zn 2*p* and W 4*f* spectra (Figure 2d,e) further verify that both elements are incorporated into the oxide lattice in fully oxidized states with no metallic features observed for any element in high‐entropy (InSnFeZnW)O_x_. The presence of several metals in different oxidation states generates a local electrostatic imbalance across the lattice, which increases the tendency for defect formation [39]. This combination of complete oxidation and heterogeneous valence states produces a defect‐tolerant lattice capable of providing electronically accessible sites [40].

### 2.3 Gas Sensing Properties Depending on Configurational Entropy

**FIGURE 3:**
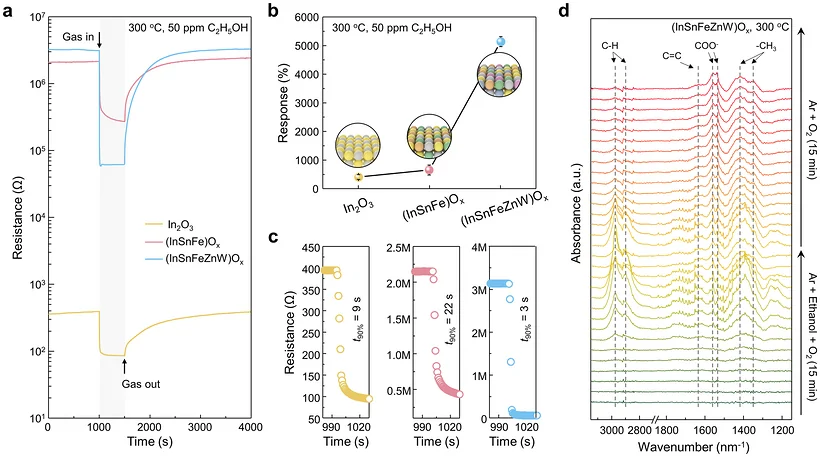
Gas sensing properties from low‐entropy to high‐entropy oxides. (a) Dynamic response curves and (b) response plot of In_2_O_3_, (InSnFe)O_x_, and (InSnFeZnW)O_x_ to 50 ppm of C_2_H_5_OH at 300°C. (c) Enlarged curves of In_2_O_3_, (InSnFe)O_x_, and (InSnFeZnW)O_x_ highlighting the response time to 50 ppm of C_2_H_5_OH at 300°C. (d) In situ DRIFTS spectra of (InSnFeZnW)O_x_ exposed to Ar (79%), O_2_ (20%), and ethanol (1%) for 15 min, followed by Ar (79%) and O_2_ (20%) for 15 min, with total flow of 1 sccm.

The synthesized In_2_O_3_, (InSnFe)O_x_, and (InSnFeZnW)O_x_ were deposited onto interdigitated electrodes (IDEs) via the drop‐casting method to fabricate gas sensors, yielding an average film thickness of 1.8 ± 0.11 µm, as determined from cross‐sectional SEM images (Figure S7). Gas sensing performances were evaluated using the customized measurement system (Figure S8). Figure 3a presents the dynamic response of sensors toward 50 ppm of ethanol at 300°C. Initially, dry air was introduced for 1000 s to stabilize the baseline resistance, followed by exposure to 50 ppm of ethanol for 500 s, and finally purging with dry air for recovery. Upon ethanol introduction, the resistance of all sensors decreases, consistent with the typical behavior of n‐type metal‐oxide gas sensors [41]. Oxygen molecules adsorb onto the metal‐oxide surface in various ionic forms depending on temperature, capturing electrons from the conduction band and forming an electronic depletion layer, as shown in Equations ((1), (2), (3)) [42].

(1)

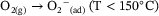

(2) $$\;O_{2\left(g\right)}+2e^{-}\to 2{O^{-}}_{\left(\mathrm{ad}\right)}\left(150^{\circ }C<T<400^{\circ }C\right)$$

(3) $$O_{2\left(g\right)}+4e^{-}\to 2{O^{2-}}_{\left(\mathrm{ad}\right)}\left(400^{\circ }C<T\right)$$

When the ethanol molecules reach the surface of the metal oxide, the ethanol reacts with the adsorbed oxygen species via a combustion‐type reaction that releases trapped electrons back to the metal oxide, as shown in Equation (4).

(4) $$C_{2}H_{5}OH_{\left(g\right)}+6{O^{-}}_{\left(\mathrm{ad}\right)}\to 2CO_{2\left(g\right)}+3H_{2}O_{\left(g\right)}+4e^{-}$$

The electron released from the surface returns to the metal oxide, resulting in a decrease in resistance. Thus, the amount of the adsorbed oxygen is a critical factor for the high response with excellent reactivity. The response of the gas sensor is defined as follows.

(5) $$\mathrm{Response}=\left(R_{a}-R_{g}\right)/R_{g}\cdot 100\left(\%\right)$$

where *R*_a_ and *R*_g_ are the resistance in air and target gas, respectively. The response toward 50 ppm of ethanol was 408% for low‐entropy In_2_O_3_, 654% for medium‐entropy (InSnFe)O_x_, and 5135% for high‐entropy (InSnFeZnW)O_x_, demonstrating an increase of gas response with configurational entropy (Figure 3b). To decouple the contribution of surface area, the response values were normalized by the BET surface area (Figure S9). The normalized responses preserve the same increasing trend, indicating that the enhancement arises from the intrinsic effects of configurational entropy. The response time, defined as the time required for the resistance to reach 90% of its total change from air to ethanol exposure, was only 3 s for (InSnFeZnW)O_x_, which is faster than that of the lower‐entropy oxides (Figure 3c). The recovery time, defined as the time required for the resistance to return 90% of baseline value after ethanol removal, was 73 s in (InSnFeZnW)O_x_, shorter than those of In_2_O_3_ (1834 s) and (InSnFe)O_x_ (2018 s). These results confirm that oxides with higher configurational entropy exhibit excellent sensitivity and faster kinetics for ethanol detection compared to lower‐entropy oxides.

In situ diffuse reflectance infrared Fourier transform spectroscopy (DRIFTS) was performed on (InSnFeZnW)O_x_ using a two‐step gas injection sequence, consisting of ethanol/Ar/O_2_ exposure followed by Ar/O_2_ purging, to investigate the reaction mechanism (Figure 3d). Upon ethanol introduction, characteristic C─H stretching bands associated with ethanol increased in intensity [43]. After cessation of ethanol flow, intermediate species were observed, including C═C stretching bands indicative of dehydration pathways, and COO^−^ stretching bands corresponding to dehydrogenation reaction [44, 45]. The coexistence of the acidic (Sn^4+^, W^6+^) and basic (Zn^2+^, In^3+^) cations within the amorphous HEO network facilitates parallel dehydration‐ and dehydrogenation‐related ethanol decomposition processes, thereby increasing the probability of kinetically favorable reaction routes [46]. Moreover, the enhanced response of HEO arises from the increased density of adsorbed oxygen and oxygen vacancies, which is further explained in detail in the mechanism section.

### 2.4 Gas‐Sensing Characteristics of the High‐Entropy (InSnFeZnW)O_x_

**FIGURE 4:**
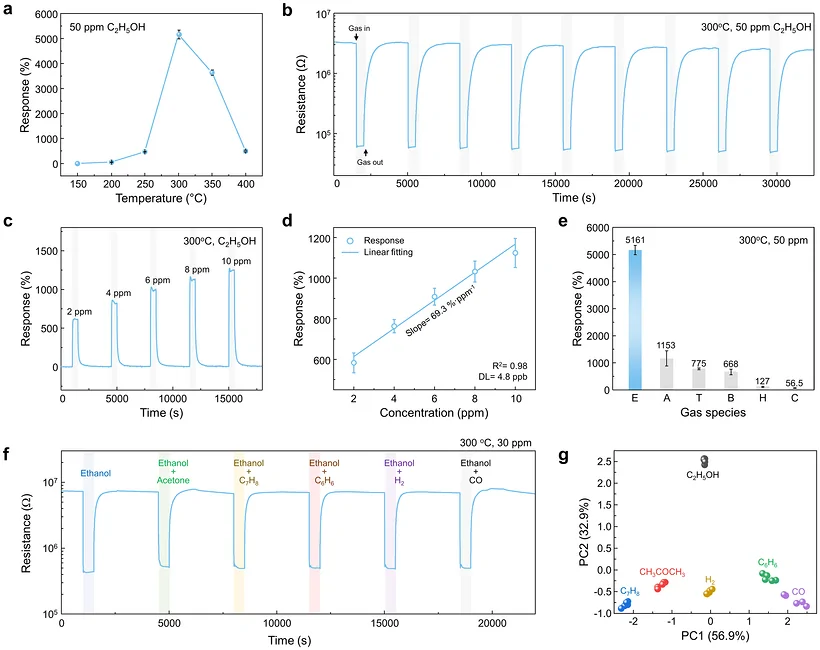
Gas sensing properties of (InSnFeZnW)O_x_. (a) Response plot of (InSnFeZnW)O_x_ to 50 ppm of C_2_H_5_OH at various operating temperatures (150‐300°C). (b) Nine consecutive pulses of 50 ppm of C_2_H_5_OH using (InSnFeZnW)O_x_ sensor at 300°C. (c) Response curves and (d) response calibration of (InSnFeZnW)O_x_ to 2–10 ppm of C_2_H_5_OH. (e) Selectivity of (InSnFeZnW)O_x_ to various gases at 300°C. (E: C_2_H_5_OH, A: CH_3_COCH_3_, T: C_7_H_8_, B: C_6_H_6_, H: H_2_, C: CO) (f) Cross selectivity (InSnFeZnW)O_x_ toward a gas mixture containing 30 ppm ethanol and interfering gases. (g) PCA for six different types of gas using (InSnFeZnW)O_x_ sensor.

The (InSnFeZnW)O_x_ HEO‐based sensor was evaluated with respect to temperature dependence, reliability, linearity, and selectivity. The responses toward 50 ppm of ethanol were measured from 150°C to 400°C (Figures 4a and S10). The response increased with temperature up to 300°C, and then decreased at higher temperatures. At low temperatures, the diffusion rate of ethanol is limited, and the adsorbed oxygen remains in the inactivated O_2_^−^ form, resulting in sluggish surface reactions. At elevated temperatures above 300°C, excessive thermal decomposition of ethanol at the upper part of the sensing layer lowers the effective reaction signal. Compared with In_2_O_3_ and (InSnFe)O_x_ over the temperature range from 150°C to 400°C, (InSnFeZnW)O_x_ exhibited the highest response at 300°C (Figure S11).

To examine the reliability of (InSnFeZnW)O_x_, the nine consecutive pulses of 50 ppm of ethanol were introduced to the (InSnFeZnW)O_x_ sensor (Figure 4b). The sensor showed repeatable and stable responses, confirming the excellent reliability of (InSnFeZnW)O_x_. To determine the linearity and detection limit (DL) of (InSnFeZnW)O_x_, sensing responses were measured over the 2–10 ppm ethanol concentration range (Figure 4c). The calibration curve revealed a slope of 69.3%·ppm^−1^ with *R* ^2^ of 0.98, indicating outstanding linearity across the tested range (Figure 4d). The theoretical DL was calculated as follows:

(6) $$R_{x^{2}}=\sum (\;{\left(y_{i}-\bar{y}\right)}^{2}$$

(7) $$\mathrm{rm}s_{\mathrm{noise}}=\sqrt{\frac{R_{x^{2}}}{N}}\;$$

(8) $$\mathrm{DL}\;=3\frac{\mathrm{rm}s_{\mathrm{noise}}}{\mathrm{slope}}$$

Here, *y_i_* represents ten data points extracted from the dynamic response curves of the (InSnFeZnW)O_x_ sensor, and $\bar{y}$ is the mean of *y_i_*. A fifth‐order polynomial fitting was used to obtain $R_{x^{2}}$. The resulting DL of the (InSnFeZnW)O_x_ sensor was 4.8 ppb, demonstrating the suitability of the HEO sensor for environmental monitoring and biomarker detection [47].

Selectivity was evaluated toward 50 ppm of ethanol, acetone (CH_3_COCH_3_), toluene (C_7_H_8_), benzene (C_6_H_6_), H_2_, and CO (Figure 4e and Figure S12). These interfering gases were selected to represent chemically similar volatile organic compounds (VOCs) that may induce cross‐sensitivity, such as acetone, toluene, and benzene, and representative reducing gases, including H_2_ and CO, which are commonly present in practical environments such as industrial processes and indoor air [48, 49, 50]. The (InSnFeZnW)O_x_ sensor exhibited the highest response to ethanol, which was about five times higher than the response to acetone, the second‐highest response gas. The cross‐selectivity of the (InSnFeZnW)O_x_ sensor was evaluated toward 30 ppm of ethanol in the presence of 30 ppm of interfering gases (Figure 4f). The ethanol response remained robust, exhibiting a relative standard deviation (RSD) of 7.4% under mixed‐gas conditions. Principal‐component analysis (PCA) was further performed using MATLAB to visualize the response patterns for various gases (Figure 4g) [51]. The PCA plot showed clear clustering and complete separation among six gases without overlap, confirming the potential for selective gas discrimination.

In addition, the humidity stability of the (InSnFeZnW)O_x_ sensor was evaluated under dry, 20%, 50%, and 80% relative humidity (RH) conditions toward 20 ppm of ethanol (Figure S13). Increasing humidity can influence ethanol diffusion and surface reactions due to competitive adsorption of water molecules, which alters the surface chemical environment of the sensing materials [52]. Across the tested RH range, the sensor exhibited an RSD of 13.9% for 20 ppm of ethanol, indicating moderate humidity‐induced variation without significant degradation of sensing functionality. The (InSnFeZnW)O_x_ also demonstrated stable and repetitive ethanol detection even under harsh conditions of 90% RH (Figure S14). Furthermore, the long‐term stability of the (InSnFeZnW)O_x_ sensors was maintained over six months (Figure S15).

Compared to previous reported ethanol sensors (Table S3), the (InSnFeZnW)O_x_ sensor demonstrated strong intrinsic ethanol‐sensing capabilities, particularly in terms of response magnitude and kinetics. While the selectivity and humidity tolerance are moderate, these values represent the intrinsic material properties without external filtering or system‐level optimization. In addition, although the optimal operating temperature is 300°C, strategies such as catalytic functionalization [53], heterostructure design [54] may provide viable pathways toward reducing the operating temperature. Further improvements in selectivity and environmental robustness can be achieved through device‐level strategies such as catalytic filtering [55], sensor arrays [1], or signal processing approaches [2].

### 2.5 Mechanism of High‐Entropy Oxide‐Based Gas Sensing Characteristics Enhancement

**FIGURE 5:**
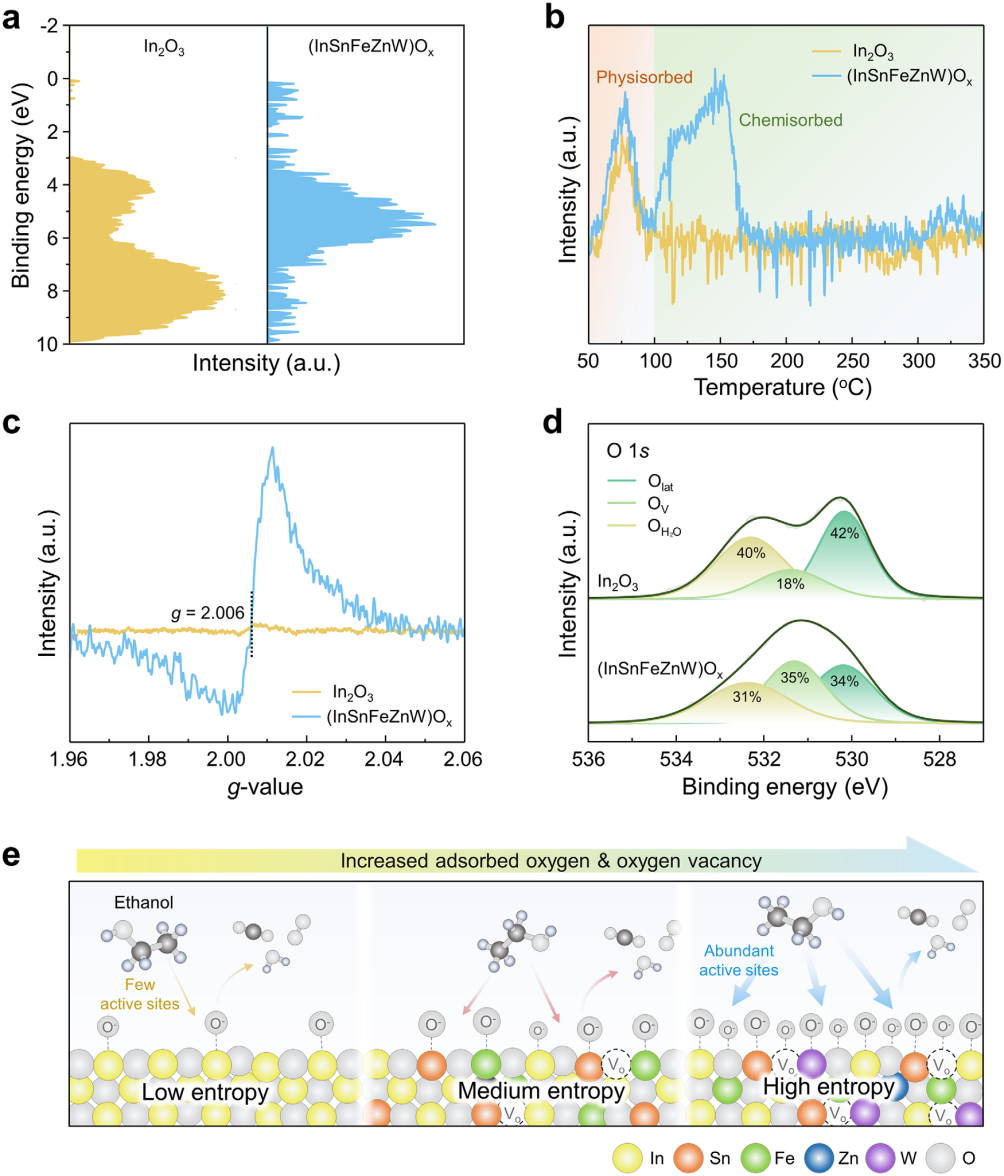
Electronic analyses supporting gas sensing enhancement in HEOs. (a) Valence band XPS spectra, (b) O_2_‐TPD, (c) ESR spectra, and (d) O 1*s* XPS spectra of In_2_O_3_ and (InSnFeZnW)O_x_. (e) Schematic illustration of low‐entropy to high‐entropy oxide‐based gas detection.

To elucidate the mechanism of the enhanced gas‐sensing properties of the high‐entropy oxide, oxygen‐adsorption behavior and defect characteristics were examined. In the valence band XPS spectra, (InSnFeZnW)O_x_ exhibits a broadened and hybridized valence feature that originates from extensive orbital mixing among the five constituent metals, whereas In_2_O_3_ displays a more localized and sharply defined valence band (Figure 5a). This broadening indicates increased electronic delocalization and diverse bonding configurations within the high‐entropy lattice [56]. Rather than implying the formation of a new electronic band, the broadened feature arises from the statistical redistribution of intrinsic valence states of the parent oxides within a single disordered lattice [57, 58, 59, 60, 61]. Additionally, the *d*‐band edge of (InSnFeZnW)O_x_ lies closer to the Fermi level, implying a strong electron‐donor character and a higher density of accessible electronic states near the Fermi level [62]. Such entropy‐driven reorganization of the valence electronic structure enhances the ability to activate adsorbed oxygen, facilitates charge transfer across the surface, and lowers the energy barrier for oxygen‐vacancy generation, producing a reactive surface environment.

O_2_‐TPD analysis provides direct evidence of the enhanced oxygen reactivity of the high‐entropy oxide (Figure 5b). The desorbed oxygen was evaluated by monitoring the ion current at m/z = 32 and integrating the signal over defined temperature ranges corresponding to different oxygen species. The total integrated O_2_‐TPD signal of (InSnFeZnW)Ox is approximately 4.88 times higher than that of In_2_O_3_, with a particularly pronounced increase in the 100–200°C region corresponding to chemisorbed oxygen (Table S4). The high‐entropy lattice supports a wider distribution of oxygen adsorption sites, which contributes to the increased chemisorption capacity. To further compare the amount of adsorbed oxygen, the resistance ratio between N_2_ and air was measured for In_2_O_3_ and (InSnFeZnW)O_x_ (Figure S16). The HEO shows a 7.9 fold larger resistance variation compared to In_2_O_3_, reflecting an increased degree of electron exchange associated with oxygen adsorption, which is directly related to gas‐sensing reactions.

Electron spin resonance (ESR) spectroscopy provides direct evidence of the increased oxygen vacancy concentration (Figure 5c). The (InSnFeZnW)O_x_ exhibits a pronounced *g* = 2.006 signal associated with paramagnetic oxygen vacancy centers, whereas In_2_O_3_ shows only a weak response. The spin concentration was further evaluated by double integration of the ESR spectra under identical measurement conditions. The integrated ESR signal of (InSnFeZnW)O_x_ is approximately 228.16 times higher than that of In_2_O_3_, indicating a significantly increased density of defect‐related unpaired electrons associated with oxygen vacancies in the high‐entropy oxide (Table S5). This enrichment of oxygen vacancies is further supported by O 1*s* XPS analysis (Figure 5d), which reveals a higher fraction of vacancy‐related and chemisorbed‐oxygen components in the high‐entropy oxide. The increased intensity of these spectral features demonstrates that configurational entropy promotes the formation and stabilization of oxygen‐related defects by accommodating local charge imbalance within the disordered lattice [40]. This defect‐rich environment increases the number of reactive surface species that contribute to adsorption and redox steps during ethanol detection (Figure 5e) [63, 64]. Taken together with the structural analyses discussed above, the high configurational entropy results in lattice‐wide disorder within the multicomponent oxide, distinguishing it from simple compositional or morphological effects.

Table S6 summarizes state‐of‐the‐art high‐entropy‐based chemoresistive gas sensors reported in the literature. Through systematic comparison of sensing performance and surface chemical analyses across different entropy levels, the present study highlights entropy‐associated modulation of surface reactivity and interfacial charge transfer, which contributes to the enhanced sensing performance of the high‐entropy oxide.

## 3 Conclusion

In summary, we demonstrated that configurational entropy provides an effective route to engineer the structural, electronic, and surface chemistry of multicomponent oxides for high‐performance ethanol sensing. The (InSnFeZnW)O_x_ high‐entropy oxide forms a compositionally uniform single phase with high configurational entropy, pronounced lattice distortion, and a large accessible surface. Entropy‐driven redistribution of electronic states shifts the *d*‐band toward the Fermi level and strengthens metal‐oxygen interactions, enabling the surface to stabilize oxygen in multiple configurations and to support a high density of vacancy‐related reactive sites. These attributes create a highly responsive interface for ethanol molecules, leading to a sensing response that is 12.6‐fold and 7.9‐fold higher than the low‐entropy and medium‐entropy oxides. The substantial improvement, supported by rapid reaction kinetics, distinct selectivity, and robust long‐term stability, highlights entropy‐engineering as a powerful strategy for designing chemoresistive sensors with enhanced reactivity and reliable long‐term performance.

## 4 Experimental Section

### 4.1 Synthesis of High‐Entropy Oxide Nanoparticles

To synthesize (InSnFeZnW)O_x_ high‐entropy oxides, the following precursors were used without further purification: In(acac)_3_ (Sigma–Aldrich, 99.99% purity), SnCl_2_ (Daejung, 98% purity), Fe(acac)_3_ (Sigma–Aldrich, 99.9% purity), Zn(acac)_2_ (Sigma–Aldrich, for synthesis), and W(CO)_6_ (Sigma–Aldrich, 97% purity). Equimolar amounts of the precursors were dissolved or suspended in anhydrous ethylene glycol to prepare a 10 mL solution with a total metal ion concentration of 0.2 M (0.04 M per metal). The solution was then transferred into a 100 mL PTFE‐lined stainless‐steel autoclave, sealed, and heated at 220°C for 20 h. After natural cooling to room temperature, the resulting precipitates were collected by centrifugation, repeatedly washed with ethanol and acetone, and dried under vacuum to obtain the HEO powders. To ensure uniform oxide formation, the as‐prepared powders were further calcined in a box furnace at 450°C for 2 h under air. The In_2_O_3_ and (InSnFe)O_x_ were synthesized using the identical process while adjusting the metal precursors according to the desired composition, maintaining the total metal ion concentration and molar ratios.

### 4.2 Gas Sensor Fabrication

IDEs were prepared by depositing 20 nm Ti and 80 nm Pt onto a p‐type Si wafer with a 300 nm SiO_2_ wet‐oxidized layer. The sensing powders (In_2_O_3_, (InSnFe)O_x_, and (InSnFeZnW)O_x_) were dispersed in deionized water of 10 g L^−1^ and sonicated for 12 h to obtain a uniform suspension. Each dispersion was drop‐cast onto the Pt/Ti IDEs in 50 µL and dried on a hot plate at 70°C after each coating cycle, repeated three times. The coated samples were annealed in a box furnace at 450°C for 2 h to improve the stability of the sensor.

### 4.3 Gas Sensor Measurement

Each sensor was wired with Pt leads through a ceramic tube and placed inside a quartz tube equipped with a tube furnace for temperature control. The electrical resistance was monitored using a source meter (Keithley 2635A) under a constant read voltage of 1 V. Gas cylinders containing 100 ppm of ethanol, acetone, toluene, benzene, H_2_, and CO (Sinjin Gas Tech) were used as analytes. Gas concentrations were adjusted with a mass flow controller by mixing the target gas with dry air while maintaining a total flow rate of 1000 sccm. Humidity levels were controlled by mixing dry air with air passed through a water bubbler, and relative humidity was calibrated at 25°C using a commercial humidity sensor.

### 4.4 Characterizations

X‐ray diffraction (XRD, D8‐Advance, BRUKER) patterns were collected in the 2θ range from 20 to 80° using an X‐ray diffractometer with Cu K_α_ radiation (λ = 1.54056 Å). A field‐emission scanning electron microscope (SEM, MERLIN Compact, ZEISS) was used to characterize the sensor. Raman spectra were acquired using a Raman spectrometer (FEX‐UP, NOST) equipped with a 532 nm Ar laser. High‐resolution transmission electron microscope (TEM, JEM‐3100F, JEOL) and energy‐dispersive X‐ray spectroscopy (EDS) mapping were employed to analyze the morphology and elemental distribution. Surface composition and chemical states were investigated by X‐ray photoelectron spectroscopy (XPS, Versaprobe III, ULVAC‐PHA) with monochromatic Al K_α_ radiation (1486.6 eV), and spectral fitting was conducted with CasaXPS software using Shirley background correction. The elemental composition of In, Sn, Fe, Zn, and W was quantified by inductively coupled plasma atomic emission spectroscopy (ICP‐AES, OPTIMA 8300, PerkinElmer). Specific surface area was obtained using BET analysis. In situ diffuse reflectance infrared Fourier transform spectroscopy (DRIFTS, Nicolet iS‐20, Thermo Scientific) measurements were performed using a mixture of sensing materials (10 mg) and KBr powder (10 mg), which was loaded into a sample cup. After loading the sample into the DRIFTS cell, pretreatment was carried out at 450°C for 2 h under flowing Ar. For ethanol DRIFTS measurements, a gas mixture of 20% O_2_ and 80% Ar was first introduced to stabilize the system and establish a baseline. Subsequently, a gas mixture of 1% ethanol, 20% O_2_, and 79% Ar was introduced and maintained at 300°C for 15 min to initiate the ethanol reaction. Finally, a mixture of 20% O_2_ and 80% Ar was supplied for an additional 15 min to monitor the decomposition of ethanol. Oxygen temperature‐programmed desorption (O_2_‐TPD) measurements were performed using a quadrupole mass spectrometer (GSD 320, Pfeiffer Vacuum). The powder samples were pelletized and sieved to obtain a particle size fraction of 200–1000 µm. Subsequently, 300 mg of the sieved sample was loaded into a quartz tube reactor with an inner diameter of 4 mm. Prior to measurement, the sample was pretreated at 300°C for 3 h under flowing Ar. After cooling to 50°C, O_2_ was introduced for 1 h to allow oxygen adsorption, followed by purging with Ar for 1 h to remove gas‐phase oxygen. The temperature was then ramped to 500°C at a heating rate of 5°C min^−1^. The effluent gas was monitored with a sampling interval of 200 ms. Mass‐to‐charge ratios (m/z) of 40 and 32 were monitored to detect Ar and O_2_, respectively. All inlet gas flow rates were maintained at 50 mL min^−1^. Oxygen vacancy concentrations of the nanoparticles were characterized by electron spin resonance (ESR, EMXplus‐9.5/12/P/L).

## Conflicts of Interest

The authors declare no conflict of interest.

## Supporting information




**Supporting File**: advs75724‐sup‐0001‐SuppMat.docx.

## Data Availability

The data that support the findings of this study are available on request from the corresponding author. The data are not publicly available due to privacy or ethical restrictions.
