# Supplementary material for: A High‐Entropy Strategy for Chemoresistive Ethanol Sensors With Remarkably Rapid and Selective Response
Source: Adv Sci (Weinh). 2026 May 20;13(45):e75724. doi: 10.1002/advs.75724 (PMC13336045; doi:10.1002/advs.75724)
Supplement: Supplementary file 1 [file ADVS-13-e75724-s001.docx]

**Supplementary Information**

**A High-Entropy Strategy for Chemoresistive Ethanol Sensors with Remarkably Rapid and Selective Response**

*Gi Baek Nam^†^, Jin Ho Seo^†^, Youngmin Kim, Hyuk Jin Kim, Yeong Jae Kim, Seon Ju Park, YunKyung Kim, Hyeon-Min Yu, Jiwoo Lee, Ji Hyeon Lim, Jiheon Lim, Jong Hun Kang, WooChul Jung, Jong Jin Jung, Jeong Woo Han, Seung Eon Moon, Seong Ju Hwang, Jong-Hoon Kang*, Zhigang Zhu*, and Ho Won Jang**

G. B. Nam, J. H. Seo, Y. Kim, H. J. Kim, Y. J. Kim, S. J. Park, Y. Kim, H.-M. Yu, J. Lee, J. Lim, J. H. Kang, W. Jung, J. W. Han, and H. W. Jang

Department of Materials Science and Engineering, Research Institute of Advanced Materials, Seoul National University, Seoul 08826, Republic of Korea

Email: hwjang@snu.ac.kr

J. H. Lim, J. H. Kang

School of Chemical and Biological Engineering and the Institute of Chemical Process, Seoul National University, Seoul 08826, Republic of Korea

J. J. Jung

Sentech Korea Corp., Paju, Gyeonggi 10880, Republic of Korea

S. E. Moon

Smart Materials Research Section, Electronics and Telecommunications Research Institute, 218 Gajeong-ro Yuseong-gu, Daejeon 34129, Republic of Korea

S. J. Hwang, J.-H. Kang

Department of Materials Science and Engineering, Pohang University of Science and Technology (POSTECH), Pohang 37673, Republic of Korea

Email: jkang@postech.ac.kr

S. J. Hwang, J.-H. Kang

Center for Van der Waals Quantum Solids, Institute for Basic Science (IBS), Pohang 37673, Republic of Korea

Email: jkang@postech.ac.kr

Zhigang Zhu

School of Health Science and Engineering, University of Shanghai for Science and Technology, Shanghai 200093, China

Email: zhigang_zhu259@163.com

H. W. Jang

Advanced Institute of Convergence Technology, Seoul National University, Suwon 16229, Republic of Korea

Email: hwjang@snu.ac.kr

^†^ These authors equally contributed to this work.

**Keywords: High-entropy oxide, Nanostructure, Solvothermal synthesis, Gas sensors, Ethanol**

**Figure S1.** Schematic illustration depicting the solvothermal synthesis mechanism of (InSnFeZnW)O_x_.

**Figure S2.** Elemental distribution of (InSnFeZnW)O_x_.

**Table S1.** Atomic percentage of constituent elements in the sample.

|  | Element | In | Sn | Fe | Zn | W |
| --- | --- | --- | --- | --- | --- | --- |
| Sample |  |  |  |  |  |  |
| In_2_O_3_ | | 100 | - | - | - | - |
| (InSnFe)O_x_ | | 27.0 | 61.1 | 11.9 | - | - |
| (InSnFeZnW)O_x_ | | 26.1 | 19.2 | 23.1 | 16.5 | 15.1 |

**Figure S3.** TEM image of (InSnFeZnW)O_x_.

**Figure S4.** TEM and STEM-EDS element mapping images of a) In and O on In_2_O_3_ and b) In, Sn, Fe, and O on (InSnFe)O_x_.

**Figure S5.** Brunauer-Emmett-Teller (BET) analysis of a) In_2_O_3_, b) (InSnFe)O_x_, and c) (InSnFeZnW)O_x_. d) Comparison of specific surface area values of reported high-entropy oxides.^[1-20]^

**Figure S6.** XPS wide scan spectra of In_2_O_3_, (InSnFe)O_x_, and (InSnFeZnW)O_x_.

**Table S2.** Binding energies of In 3*d*, Sn 3*d*, and Fe 2*p* in In_2_O_3_, (InSnFe)O_x_, and (InSnFeZnW)O_x_.

|  | In 3*d*_5/2_ (eV) | Sn 3*d*_5/2_ (eV) | Fe 2*p*_3/2_ (eV) |
| --- | --- | --- | --- |
| In_2_O_3_ | 444.84 |  |  |
| (InSnFe)O_x_ | 445.02 | 486.78 | 711.08 |
| (InSnFeZnW)O_x_ | 445.93 | 487.27 | 711.22 |

**Figure S7.** a, b) SEM images of (InSnFeZnW)O_x_ deposited on 5 µm-gap IDEs. c-f) Cross-sectional SEM image of (InSnFeZnW)O_x_ showing the thickness of the deposition layer.

**Figure S8.** Schematic illustration of the gas sensing measurement setup.

**Figure S9.** Response normalized by the BET specific surface area for In_2_O_3_, (InSnFe)O_x_, and (InSnFeZnW)O_x_ toward 50 ppm of C_2_H_5_OH at 300 °C, defined as the sensor response divided by the specific surface area of each material.

**Figure S10.** Dynamic response of (InSnFeZnW)O_x_ to 50 ppm of C_2_H_5_OH at different operating temperatures: a) 150 °C, b) 200 °C, c) 250 °C, d) 300 °C, e) 350 °C, and f) 400 °C.

**Figure S11.** Dynamic response of In_2_O_3_ to 50 ppm of C_2_H_5_OH at different operating temperatures: a-1) 150 °C, a-2) 200 °C, a-3) 250 °C, a-4) 300 °C, a-5) 350 °C, and a-6) 400 °C. a-7) Response plot of In_2_O_3_ to 50 ppm of C_2_H_5_OH at various operating temperatures (150-300 °C). Dynamic response of (InSnFe)O_x_ to 50 ppm of C_2_H_5_OH at different operating temperatures: b-1) 150 °C, b-2) 200 °C, b-3) 250 °C, b-4) 300 °C, b-5) 350 °C, and b-6) 400 °C. b-7) Response plot of (InSnFe)O_x_ to 50 ppm of C_2_H_5_OH at various operating temperatures (150-300 °C).

**Figure S12.** Dynamic response of (InSnFeZnW)O_x_ to 50 ppm of different gases: a) C_2_H_5_OH, b) CH_3_COCH_3_, c) H_2_, d) C_6_H_6_, e) C_7_H_8_, and f) CO.

**Figure S13.** Dynamic curves of (InSnFeZnW)O_x_ to 20 ppm of C_2_H_5_OH under different humid conditions: a) dry, b) 20% RH, c) 50% RH, and d) 80% RH. e) Response plot of (InSnFeZnW)O_x_ to C_2_H_5_OH under various humid conditions.

**Figure S14.** Repetitive curves of (InSnFeZnW)O_x_ to 10 ppm of C_2_H_5_OH under 90% RH condition.

**Figure S15.** a) One-month, b) three-month, and c) six-month stability of (InSnFeZnW)O_x_ to 50 ppm of ethanol at 300 °C. d) Response plot of (InSnFeZnW)O_x_ over storage period.

**Table S3.** Comparison of gas sensing performance of chemoresistive ethanol sensors

| **Materials** | **Operating**  **temperature** | **Concentration** | **Response**  **(**(*R*_a_-*R*_g_)/*R*_g_·100%**)** | **Detection limit** | **Response**  **time** | **Recovery**  **time** | **Long-term duration** | **Ref** |
| --- | --- | --- | --- | --- | --- | --- | --- | --- |
| (InSnFeZnW)O_x_ | 300 °C | 50 ppm | 5,135% | 4.8 ppb | 3 s | 72 s | 6 months | This work |
| Al-doped NiO nanorod-flowers | 200 °C | 100 ppm | 1,200% | - | 48 s | 40 s | - | [21] |
| ZnO/SnO_2_ hollow spheres | 225 °C | 30 ppm | 3,480% | 500 ppb | 1 s | 120 s | 30 days | [22] |
| SnO_2_/α-Fe_2_O_3_ hollow nanofiber | 340 °C | 100 ppm | 2,037% | 2 ppm | 5 s | 14 s | 5 days | [23] |
| Fe_2_O_3_@SnO_2_ nanospindle | 320 °C | 100 ppm | 280% | 2 ppm | 5 s | 5 s | - | [24] |
| CeO_2_/SnO_2_ composites | 225 °C | 100 ppm | 3,700% | 10 ppm | 2 s | 70 s | - | [25] |
| W-doped In_2_O_3_ nanoparticles | 275 °C | 100 ppm | 3,320% | - | 10 s | 60 s | 50 days | [26] |
| ZnO@In_2_O_3_ nanofibers | 225 °C | 100 ppm | 1,800% | 5 ppm | 3.7 s | 52.0 s | 30 days | [27] |
| Bi_2_O_3_-decorated In_2_O_3_ | 200 °C | 200 ppm | 1,770% | - | 24 s | 180 s | - | [28] |
| TiO_2_/Ag_0.35_V_2_O_5_ | 350 °C | 50 ppm | 1,500% | - | 7 s | 8 s | - | [29] |
| ZnS/In_2_O_3_ | 250 °C | 100 ppm | 1,170% |  | 21 s | 34 s | 32 days | [30] |
| SnO_2_-ZnO | 250 °C | 100 ppm | 3,000% | 0.2 ppm | 3 s | 45 s | 15 days | [31] |
| Au-ZnO nanorods | 270 °C | 100 ppm | 68.9% | - | 3.39 s | 179.38 s | 28 days | [32] |
| Pd-ZnO nanorods | 260 °C | 500 ppm | 512% | - | 6 s | 95 s | 22 days | [33] |
| WO_3_ nanoflakes | 250 °C | 50 ppm | 2,050% | - | 580 s | 1,260 s | 14 days | [34] |

**Table S4.** Semi-quantitative integration of O_2_-TPD signals over different temperature ranges corresponding to physisorbed (50-100 ^°^C) and chemisorbed (100-200 ^°^C) oxygen species for In_2_O_3_ and (InSnFeZnW)O_x_.

| Temperature (^o^C)  Area (a.u.) | 50-100  (Physisorbed) | 100-200  (Chemisorbed) | 50-200  (Total) |
| --- | --- | --- | --- |
| In_2_O_3_ | 5.06×10^-13^ | 3.50×10^-14^ | 5.06×10^-13^ |
| (InSnFeZnW)O_x_ | 7.88×10^-13^ | 1.85×10^-12^ | 2.64×10^-12^ |

The total integrated area (50-200 ^°^C) of (InSnFeZnW)O_x_ is 4.88 times higher than that of In_2_O_3_, with a particularly pronounced increase in the 100-200 ^°^C region associated with reactive chemisorbed oxygen.

**Figure S16.** Resistance ratio between N_2_ and air atmospheres of In_2_O_3_ and (InSnFeZnW)O_x_.

**Table S5.** Double-integrated ESR signal intensities (DI) of In_2_O_3_ and (InSnFeZnW)O_x_.

| Sample | In_2_O_3_ | (InSnFeZnW)O­_x_ |
| --- | --- | --- |
| DI (a.u.) | 1.12×10^-14^ | 2.56×10^-12^ |

| $c$ | point sample calibration factor |
| --- | --- |
| $B_{1}$ | microwave magnetic field |
| $B_{m}$ | modulation amplitude |
| $f(B_{1},B_{m})$ | spatial distribution of $B_{1}$ and $B_{m}$ |
| $G_{R}$ | receiver gain |
| $C_{t}$ | conversion time |
| $n$ | number of averages |
| $\sqrt{P}$ | square root of the microwave power |
| $Q$ | quality factor of the resonator |
| $n_{B}$ | Boltzmann factor to correct for temperature |
| $S$ | electronic spin |
| $n_{S}$ | number of spins |

The number of spins ($n_{S}$) can be expressed as:

$$n_{S}= \frac{\mathrm{DI}}{\frac{c}{f(B_{1},B_{m})}\times\left\{ G_{R}\times C_{t}\times n \right\}\times\{\sqrt{P}\times B_{m}\times Q\times n_{B}\times S\times\left( S+1 \right)\}}$$

Since all measurement conditions were kept identical for all samples, the experimental parameters remain constant. Therefore, the ratio of spin concentrations simplifies to:

$$\frac{n_{S, (InSnFeZnW)O_{x}}}{n_{S, \mathrm{In}_{2}O_{3}}}=\frac{\mathrm{DI}_{(InSnFeZnW)O_{x}}}{\mathrm{DI}_{\mathrm{In}_{2}O_{3}}}$$

**Table S6.** State-of-the-art high-entropy-based chemoresistive gas sensors.

| **Materials** | **Synthesis methods** | **Gas species** | **Concentration** | **Response**  **(**(*R*_a_-*R*_g_)/*R*_g_·100%**)** | **Mechanism**  **investigation** | **Ref** |
| --- | --- | --- | --- | --- | --- | --- |
| (InSnFeZnW)O_x_ | Solvothermal method | Ethanol | 50 ppm | 5,135% | Systematic experimental comparison | This work |
| TiZrCrVNi-MoS_2_ | Arc melting | Trimethylamine | 2000 ppm | 400% | Not investigated | [35] |
| AlCoFeNiCu-quasicrystal | Chemical exfoliation | NO_2_ | 100 ppm | 46% | Not investigated | [36] |
| AlCoFeNiCu-MoS_2_ | Arc melting | NO_2_ | 100 ppm | 66% | DFT calculations | [37] |
| AgCuPdAuPt-MoS_2_ | Arc melting | H_2_ | 1000 ppm | 32% | DFT calculations | [38] |
| Gd_0.2_La_0.2_Ce_0.2_Hf_0.2_Zr_0.2_O_2_ | Co-precipitation | CH_4_ | 100 ppm | 48.28% | Not investigated | [39] |
| Ga, Ni, Mn, Cr, Ru, La, Fe perovskite structure | Hydrothermal method | CH_4_ | 25 ppm | 54% | DFT calculations | [40] |
| MnFeCoNiCu-SnO_2_ | Oil-phase synthesis / wet impregnation | Acetone | 0.5 ppm | 417% | Not investigated | [41] |
| PtFeCoNiCuSn-SnO_2_ | Wet-chemical synthesis | Acetone | 2 ppm | 459% | DFT calculations | [42] |

**References**

[1]	K. Gu, D. Wang, C. Xie, T. Wang, G. Huang, Y. Liu, Y. Zou, L. Tao, S. Wang, *Angewandte Chemie* **2021**, 133, 20415.

[2]	Y. Wang, H. He, H. Lv, F. Jia, B. Liu, *Nature Communications* **2024**, 15, 6761.

[3]	D. Feng, Y. Dong, L. Zhang, X. Ge, W. Zhang, S. Dai, Z. A. Qiao, *Angewandte Chemie* **2020**, 132, 19671.

[4]	S. Qi, Z. Lei, Q. Huo, J. Zhao, T. Huang, N. Meng, J. Liao, J. Yi, C. Shang, X. Zhang, *Advanced Materials* **2024**, 36, 2403958.

[5]	Z. Meng, X. Gong, J. Xu, X. Sun, F. Zeng, Z. Du, Z. Hao, W. Shi, S. Yu, X. Hu, *Chemical Engineering Journal* **2023**, 457, 141242.

[6]	T. X. Nguyen, C.-C. Tsai, J. Patra, O. Clemens, J.-K. Chang, J.-M. Ting, *Chemical Engineering Journal* **2022**, 430, 132658.

[7]	X. Duan, X. Wang, L. Xu, T. Ma, Y. Shu, S. Hou, Q. Niu, P. Zhang, *Journal of Materials Chemistry A* **2023**, 11, 19696.

[8]	T. X. Nguyen, C.-H. Lee, J.-H. Sun, C.-K. Peng, W.-H. Chu, H. Pourzolfaghar, Y.-R. Lin, M. Ghufron, Y.-Y. Li, Y.-H. Chang, *Chemical Engineering Journal* **2025**, 511, 161731.

[9]	X. Miao, Z. Peng, L. Shi, S. Zhou, *ACS Catalysis* **2023**, 13, 3983.

[10]	H. Su, C. Ma, K. Zhou, C. Zhu, L. Tang, L. Luo, X. Wang, D. Zeng, *Chemical Engineering Journal* **2025**, 509, 161377.

[11]	S. C. Karthikeyan, S. Ramakrishnan, S. Prabhakaran, M. R. Subramaniam, M. Mamlouk, D. H. Kim, D. J. Yoo, *Small* **2024**, 20, e2402241.

[12]	C. Feng, Y. Zhou, Z. Xie, Z. Yang, L. Zou, P. Wang, W. Lian, P. Xiaokaiti, Y. Kansha, A. Abudula, *Chemical Engineering Journal* **2024**, 495, 153408.

[13]	H. Yang, L. He, Q. Chen, J. Zhu, G. Jiang, N. Qiu, Y. Wang, *Chemical Engineering Journal* **2024**, 488, 151113.

[14]	P. Tukur, Y. Wei, Y. Zhang, H. Chen, Y. Lin, S. He, Y. Mo, J. Wei, *Small* **2025**, 21, 2501946.

[15]	H. Min, C. Kim, S. Y. Lin, J. Choi, Y. Sim, B. Y. Yu, J. H. Moon, *Advanced Materials* **2025**, 37, 2418767.

[16]	Y. Tao, L. Wang, T. Zhang, C. Wang, S. Cui, B. Liu, *Small* **2025**, 21, 2500962.

[17]	H. Nan, S. Lv, Z. Xu, Y. Feng, Y. Zhou, M. Liu, T. Wang, X. Liu, X. Hu, H. Tian, *Chemical Engineering Journal* **2023**, 452, 139501.

[18]	C. Xu, H. Mou, D. Li, D. Zhang, L. Feng, S. Lv, C. Song, S. Sun, J. Song, D. Wang, *Applied Catalysis B: Environment and Energy* **2025**, 371, 125248.

[19]	X. Liu, R. Tao, C. Li, J. Wang, S. Yao, C. Hong, H. Li, J. Geng, J. Liang, *Chemical Engineering Journal* **2024**, 484, 149791.

[20]	C. Hong, R. Tao, S. Tan, L. A. Pressley, C. A. Bridges, H. Y. Li, X. Liu, H. Li, J. Li, H. Yuan, *Advanced Functional Materials* **2025**, 35, 2412177.

[21]	C. Wang, X. Cui, J. Liu, X. Zhou, X. Cheng, P. Sun, X. Hu, X. Li, J. Zheng, G. Lu, *ACS Sensors* **2016**, 1, 131.

[22]	J. Liu, T. Wang, B. Wang, P. Sun, Q. Yang, X. Liang, H. Song, G. Lu, *Sensors and Actuators B: Chemical* **2017**, 245, 551.

[23]	B. Wang, X. Fu, F. Liu, S. Shi, J. Cheng, X. Zhang, *Journal of alloys and compounds* **2014**, 587, 82.

[24]	X. Liu, J. Zhang, X. Guo, S. Wang, S. Wu, *RSC Advances* **2012**, 2, 1650.

[25]	J. Liu, M. Dai, T. Wang, P. Sun, X. Liang, G. Lu, K. Shimanoe, N. Yamazoe, *ACS applied materials & interfaces* **2016**, 8, 6669.

[26]	Z. Jin, Z. Zhao, G. Jin, Z. Shao, L. Wu, *Ceramics International* **2025**, 51, 10423.

[27]	B. Huang, Z. Zhang, C. Zhao, L. Cairang, J. Bai, Y. Zhang, X. Mu, J. Du, H. Wang, X. Pan, *Sensors and Actuators B: Chemical* **2018**, 255, 2248.

[28]	S. Park, S. Kim, G.-J. Sun, C. Lee, *ACS applied materials & interfaces* **2015**, 7, 8138.

[29]	Y. Wang, L. Liu, C. Meng, Y. Zhou, Z. Gao, X. Li, X. Cao, L. Xu, W. Zhu, *Scientific Reports* **2016**, 6, 33092.

[30]	Q. Chen, S. Ma, X. Xu, H. Jiao, G. Zhang, L. Liu, P. Wang, D. Gengzang, H. Yao, *Sensors and Actuators B: Chemical* **2018**, 264, 263.

[31]	B. Jiang, T. Zhou, L. Zhang, J. Yang, W. Han, Y. Sun, F. Liu, P. Sun, H. Zhang, G. Lu, *Sensors and Actuators B: Chemical* **2023**, 393, 134257.

[32]	Y.-L. Chu, S.-J. Young, Y.-R. Huang, S. Arya, T.-T. Chu, *ACS Applied Electronic Materials* **2025**, 7, 2327.

[33]	P. Cao, Z. Yang, S. Navale, S. Han, X. Liu, W. Liu, Y. Lu, F. Stadler, D. Zhu, *Sensors and Actuators B: Chemical* **2019**, 298, 126850.

[34]	E. Spagnoli, S. Krik, B. Fabbri, M. Valt, M. Ardit, A. Gaiardo, L. Vanzetti, M. Della Ciana, V. Cristino, G. Vola, *Sensors and Actuators B: Chemical* **2021**, 347, 130593.

[35]	S. S. Mishra, K. U. Mb, S. P. Thomas, C. S. Tiwary, K. Biswas, V. B. Kamble, *ACS applied materials & interfaces* **2022**, 14, 13653.

[36]	S. Mishra, S. Kumar, P. Kumbhakar, N. K. Katiyar, R. Tromer, C. F. Woellner, D. S. Galvao, C. S. Tiwary, M. Kumar, K. Biswas, *Materials Chemistry and Physics* **2023**, 298, 127449.

[37]	S. Kumar, R. Chaurasiya, S. S. Mishra, P. Kumbhakar, G. Meng, C. S. Tiwary, K. Biswas, M. Kumar, *ACS Applied Nano Materials* **2023**, 6, 5952.

[38]	K. M. Urs, N. K. Katiyar, R. Kumar, K. Biswas, A. K. Singh, C. Tiwary, V. Kamble, *Nanoscale* **2020**, 12, 11830.

[39]	V. R. Naganaboina, M. Anandkumar, A. S. Deshpande, S. G. Singh, *Sensors and Actuators B: Chemical* **2022**, 357, 131426.

[40]	Z. Luo, X.-P. Zhou, *Science Advances* **2025**, 11, eadw1461.

[41]	O. Wang, Z. Ma, Z. Xue, M. Yan, B.-L. An, Y. Zhao, J. Xu, X. Wang, *ACS nano* **2025**, 19, 13325.

[42]	O. Wang, H. Wang, Y. Tang, Z. Ma, B.-L. An, Y. Zhao, X. Wang, J. Xu, *ACS nano* **2025**, 19, 42466.
